# Drug resistant typhoid fever: A clinical challenge and a potential solution

**DOI:** 10.12669/pjms.40.7.10052

**Published:** 2024-08

**Authors:** Muhammad Aliyan Ahmed, Yasir Ahmad, Muhammad Saad

**Affiliations:** 1 Muhammad Aliyan Ahmed, MBBS, Liaquat University of Medical and Health Sciences, Jamshoro, Sindh, Pakistan; 2 Yasir Ahmad, MBBS, Saidu Medical College, Swat, KPK, Pakistan; 3 Muhammad Saad, MBBS, Liaquat University of Medical and Health Sciences, Jamshoro, Sindh, Pakistan

Typhoid Fever is a lethal bacterial infection caused by Salmonella typhi bacteria. It is characterized by high grade fever, abdominal pain, severe toxicity, sore throat, tongue ulcers, and either diarrhea or constipation. Although antibiotics are the first line treatment, but over the years, the bacteria have developed resistance to these drugs. MDR (Multi-Drug Resistant) S. typhi strains are resistant to at least one of three commonly used antimicrobials, including ampicillin, sulfonamides, and chloramphenicol. On the other hand, XDR (Extensively Drug Resistant) strains are only sensitive to piperacillin/tazobactam, azithromycin, and carbapenems.^1^ In Pakistan, the rate of Typhoid is estimated to be the highest among South Asian countries, with 493.5 cases per 100,000. A recent 2021 study conducted in a tertiary care hospital in Lahore revealed that XDR typhoid accounted for 46.1% of cases, while MDR typhoid accounted for another 25%.^1^ In view of these observations, and the fact that we overuse antibiotics, it is only logical to expect the bacteria to develop resistance against the remaining antibiotics, and it is important that we explore other treatment options.

Phage Therapy employs the use of bacteriophage to kill bacteria. It was popular in the first half of 20th century, however due to development of antibiotics, interest in using phages for therapeutic purposes stalled.^2^ In 1946, a Los Angeles case series showcased the significant impact of bacteriophages in treating typhoid fever.

Another study states that administering isolated bacteriophages and antibiotics simultaneously had a synergistic effect on reducing the bacterial load in laboratory settings. Also this synergistic effect showed to decrease antibiotic resistance.^3^ The bacterial killing mechanism and resistance development differ between phages and antibiotics. Phages evolve alongside bacteria, preventing resistance. This unique trait enables phage therapy to target drug-resistant strains effectively. In summary, phage therapy is safe, host specific, highly efficacious, and cost-effective form of treatment.^2^

To combat antibiotic-resistant Typhoid strains effectively, it is imperative to explore and revitalize phage therapy. Establishing research centers and conducting large-scale clinical trials in Pakistan would elucidate its efficiency against MDR and XDR strains.
